# Chronic Exposure to Arsenic in the Drinking Water Alters the Expression of Immune Response Genes in Mouse Lung

**DOI:** 10.1289/ehp.0800199

**Published:** 2009-03-04

**Authors:** Courtney D. Kozul, Thomas H. Hampton, Jennifer C. Davey, Julie A. Gosse, Athena P. Nomikos, Phillip L. Eisenhauer, Daniel J. Weiss, Jessica E. Thorpe, Michael A. Ihnat, Joshua W. Hamilton

**Affiliations:** 1 Department of Pharmacology and Toxicology, Dartmouth Medical School, Hanover, New Hampshire, USA; 2 Center for Environmental Health Sciences, Dartmouth College, Hanover, New Hampshire, USA; 3 Vermont Lung Center, University of Vermont College of Medicine, Burlington, Vermont, USA; 4 Department of Cell Biology, University of Oklahoma Health Sciences Center, Oklahoma City, Oklahoma, USA

**Keywords:** arsenic, inflammation, innate immune system, lung, migration

## Abstract

**Background:**

Chronic exposure to drinking water arsenic is a significant worldwide environmental health concern. Exposure to As is associated with an increased risk of lung disease, which may make it a unique toxicant, because lung toxicity is usually associated with inhalation rather than ingestion.

**Objectives:**

The goal of this study was to examine mRNA and protein expression changes in the lungs of mice exposed chronically to environmentally relevant concentrations of As in the food or drinking water, specifically examining the hypothesis that As may preferentially affect gene and protein expression related to immune function as part of its mechanism of toxicant action.

**Methods:**

C57BL/6J mice fed a casein-based AIN-76A defined diet were exposed to 10 or 100 ppb As in drinking water or food for 5–6 weeks.

**Results:**

Whole genome transcriptome profiling of animal lungs revealed significant alterations in the expression of many genes with functions in cell adhesion and migration, channels, receptors, differentiation and proliferation, and, most strikingly, aspects of the innate immune response. Confirmation of mRNA and protein expression changes in key genes of this response revealed that genes for interleukin 1β, interleukin 1 receptor, a number of toll-like receptors, and several cytokines and cytokine receptors were significantly altered in the lungs of As-exposed mice.

**Conclusions:**

These findings indicate that chronic low-dose As exposure at the current U.S. drinking-water standard can elicit effects on the regulation of innate immunity, which may contribute to altered disease risk, particularly in lung.

Chronic exposure to arsenic is a significant worldwide environmental health concern (Agency for Toxic Substances and Disease Registry 1999; National Research Council 1999). The primary route of exposure is through drinking water that has been contaminated by natural geologic sources of As. In recognition of the health risks associated with chronic As exposure, the U.S. Environmental Protection Agency (EPA) recently reduced its drinking-water standard in regulated public water sources from 50 ppb to 10 ppb (0.67–0.13 μM), but this standard does not cover private, unregulated wells (U.S. EPA 2001). Thus, drinking-water As exposure remains an important public health concern in many areas of the United States, such as New Hampshire, where as much as half of the population acquires their water from private wells and where As is naturally found at levels higher than the federal guidelines in a significant fraction of these wells (Karagas et al. 2002). Similar scenarios can be found in many other areas of the United States and throughout the world. Through mechanisms that remain unclear, chronic exposure to As has been associated with many diseases, including lung, liver, skin, kidney, and bladder cancer, cardiovascular disease, and diabetes (Abernathy et al. 1999; National Research Council 1999; Smith et al. 1992; Tapio and Grosche 2006; Watanabe et al. 2003). Drinking-water As has also been implicated in impaired lung function, bronchiectasis, and increased risks of respiratory illness (Ghosh et al. 2007; Smith et al. 2006). As a lung toxicant, As may be unique in its ability to increase the risk of these various lung diseases via ingestion rather than inhalation.

Many reports indicate that As can have significant effects on many aspects of the immune system in experimental systems, including suppression of contact hypersensitivity responses (Patterson et al. 2004), increased interleukin-1α (IL-1α) expression (Corsini et al. 1999), altered expression of cytokines such as granulocyte-macrophage colony-stimulating factor (GM-CSF) (Vega et al. 2001), loss of adhesion, impairment of function, and morphologic changes in human macrophages (Lage et al. 2006; Lemarie et al. 2006). Much of the *in vitro* and *in vivo* work has been done at As doses well above the current U.S. EPA standard, which are not necessarily relevant to U.S. exposure levels. However, a recent study in zebrafish (*Danio rerio*) reported that exposures to very low doses (2 and 10 ppb) could elicit significant effects on innate immune mediators, such as decreases in interleukin-1β gene (*Il1b*) mRNA after bacterial infection and increases in bacterial and viral loads (Nayak et al. 2007). Epidemiologic data also have suggested that chronic As exposure can alter immune function, including reports of defective IL-2 receptor (IL2R) expression in the lymphocytes of patients with As-induced Bowen’s disease (Yu et al. 1998), altered expression of inflammatory markers and immune mediators in lymphocytes of As-exposed adults (Andrew et al. 2008; Wu et al. 2003), and altered activation of T-cell processes and increased GM-CSF secretion in children exposed to As in drinking water (Soto-Pena et al. 2006). Although a specific link between As exposure and immune dysfunction had not been identified to date, many of the diseases associated with chronic As exposure, such as diabetes and cancer, often have strong immune- mediated components. There has been a recent call for more intensive studies on the immunotoxic effects of exposure to environmental toxicants, such as As, and the role of such responses in disease risk (Selgrade 2007).

In a previous study, we reported that As in drinking water profoundly affected gene expression in mouse lung at very low doses, at or below the current U.S. drinking-water standard, using whole genome transcriptome profiling (Andrew et al. 2007). From the patterns of the responses in that study, we hypothesized that immune modulation, particularly of the innate immune system, may be one aspect to the As response in lung. The innate immune response is the body’s first mechanism for defense against pathogens and is capable of inducing inflammation and, if necessary, triggering the adaptive immune response. Innate immunity has multiple chemical, physical, and biological first-line defense mechanisms, some of which include cytokines, the complement system, toll-like receptors (TLRs), and interferon. Our goal in the present study was to investigate this hypothesis at the mRNA level, using an advanced microarray chip and rank-product statistical analysis, which identifies gene sets of greater biological relevance. In addition, we aimed to confirm the microarray results with protein and cellular changes.

## Materials and Methods

### Animal husbandry

All animal studies were conducted in accordance with guidelines approved by the Association for Assessment and Accreditation of Laboratory Animal Care using a protocol approved by the Institutional Animal Care and Use Committee at the University of Oklahoma Health Sciences Center, Dartmouth Medical School, and the University of Vermont College of Medicine. All animals were treated humanely and with regard for alleviation of suffering.

For the microarray analysis, we housed 6-week-old C57BL/6J male mice (APA breeding stock, National Cancer Institute, Frederick, MD; *n* = 4–6 per group) in ventilated cages with autoclaved nanopure water available *ad libitum*, autoclaved bedding, and *ad libitum* autoclaved AIN-76A chow that had been specially formulated for this study by the manufacturer, with or without addition of 10 ppb sodium arsenite (Harlan Teklad, Madison, WI). At the start of the experiment, animals were given drinking water (changed biweekly) with or without addition of 10 or 100 ppb sodium arsenite for 5 weeks. We conducted analyses with 10 ppb As added to the diet only for the microarray study. Animals were euthanized with carbon dioxide; the lungs were removed and placed in RNAlater (Ambion, Austin, TX) on ice.

For protein analysis, 7-week-old C57BL/6J male mice (Jackson Laboratories, Bar Harbor, ME; *n* = 6 per group, two experimental repeats) were housed as described above with AIN-76A chow *ad libitum*. At the start of the experiment, animals were given drinking water (changed weekly) with or without addition of 10 or 100 ppb sodium arsenite. In the first experiment, mice were maintained on As for 5 weeks, and in the second, repeat experiment, mice were maintained on As for 6 weeks. Animals were euthanized by cervical dislocation, and the lungs of animals were snap frozen. For cellular analysis of broncho-alveolar lavage fluid (BALF), mice (*n* = 5–6 per group, 5-week exposure) were euthanized with pentobarbital and BALF was collected by *in situ* cannulation of the trachea.

### BALF collection

We lavaged lungs *in situ* with 1 mL phosphate-buffered saline. We centrifuged BALF for collection of cells and obtained total cell counts with Advia 120 Hematology System (Bayer Health Care LLC, Tarrytown, NY). We used remaining cells for cytospin preparations, which we stained with PROTOCOL Hema-3 stain set (Fisher, Houston, TX). Ten random fields per slide were counted to obtain the percentage of cell differentials (macrophages, neutrophils, and lymphocytes) recovered from the BALF.

### RNA isolation and microarray

We extracted RNA from frozen lung samples or cell monolayers and homogenized it using Qiashredder and RNeasy Mini Kits (Qiagen, Valencia, CA) per manufacturer protocol. We removed contaminating genomic DNA using DNA-free kits (Ambion, Austin, TX) and quantified total RNA with an ND-1000 spectrophotometer (NanoDrop Technologies, Rockland, DE). We determined RNA quality with the RNA 6000 Nano Chip Kit (Agilent Technologies, Inc., Santa Clara, CA). We performed microarray experiments using RNA samples from the study conducted at the University of Oklahoma and GeneChip Mouse Genome 430 2.0 arrays from Affymetrix (Santa Clara, CA). Details of procedure have been previously described (Kozul et al. 2008). Briefly, we processed total RNA samples after a standard one-cycle eukaryotic target preparation protocol from Affymetrix. RNA was first reverse-transcribed using T7-oligo(dT) promoter primer in the first-strand cDNA synthesis reaction. After RNase H-mediated second-strand cDNA synthesis, we purified the double-stranded cDNA, which served as a template in the subsequent *in vitro* transcription (IVT) reaction. The IVT reaction was carried out in the presence of T7 RNA polymerase and a biotinylated nucleotide analog/ribonucleotide mix for complementary RNA (cRNA) amplification and biotin labeling. Biotinylated cRNA (15 μg) targets were then purified, fragmented, and hybridized to GeneChip array during the overnight incubation at 45°C in a rotating hybridization oven. After hybridization, we stained the arrays with streptavidin-phycoerythrin in the GeneChip Fluidics station and then scanned them using the Affymetrix GeneChip Scanner (laser filter set at 570 nm; 2.5 μm pixel size). Gene symbols are based on Entrez Gene official gene symbols. Supplemental Material, Table 1 (http://www.ehponline.org/members/2009/0800199/suppl.pdf) lists accession identification numbers (IDs).

### Microarray statistical analysis

Microarray analysis procedures used here have been previously described (Gosse et al. 2008; Kozul et al. 2008). Briefly, we identified differentially expressed genes between classes using the nonparametric rank-product method (*p* < 0.05) as previously described (Breitling and Herzyk 2005; Breitling et al. 2004) and generated log_2_ expression values for each slide. We calculated mean fold changes in R open-source programming language (R Foundation for Statistical Computing Vienna, Austria) for each probe within classes for use in subsequent filtering. We performed clustering using the Simpleaffy package in BioConductor (http://www.bioconductor.org/), using its default, standard Pearson correlation. We generated the network analysis with Ingenuity Pathway Analysis (IPA; version 5.1; Ingenuity Systems, Redwood City, CA). We did additional analyses with Affymetrix NETAFFX expression query.

### Quantitative real-time polymerase chain reaction

We isolated total RNA as described above. RNA (1 μg) was reverse-transcribed with Omniscript Reverse Transcriptase (Qiagen) and with primers that were synthesized using Oligo 6 software (Molecular Biology Insights, Cascade, CO) to specifically amplify selected genes (*Il1b*, *Il1r2*) or with random primers (*Adam10*, *Scn3b;* Integrated DNA Technologies, Coralville, IA). *Il1b* primer sequence was 5′-CTT CTC CAC AGC CAC ATT GA-3′, and *Il1r2* sequence was 5′-GCT GGA GAT GTC GGA GT-3′. We performed quantitative real-time reverse transcriptase polymerase chain reaction (RT-PCR) (*n* = 4–6 per group) to assess relative quantities of each transcript with the 7500 real-time PCR system (Applied Biosystems, Foster City, CA) using Taqman Gene Expression Assays (Applied Biosystems). We included negative controls, including samples that would detect either contaminating genomic DNA in the RNA samples or contamination of PCR reagents. We ran an internal standard curve for each transcript on each plate, consisting of a serial dilution of cDNA known to contain the transcript in question. Relative transcript levels were determined from the standard curve and normalized to the control group. To control for pipetting errors, we quantified cDNA levels with the NanoDrop ND-1000 spectrophotometer after reverse transcription with random primers, which did not vary between samples.

### Immunoblot analysis

Whole lung tissue (left lobe) was homogenized in 400 mL lysis buffer [50 mM Tris-HCl (pH 8.0), 100 mM NaCl, 10% NP-40 detergent] with protease inhibitors phenylmethylsulfonyl fluoride (Sigma, St. Louis, MO), aprotinin (Sigma), and leupeptin (Sigma), rocked at 4°C for 15 min, and centrifuged at 15,000 rpm at 4°C for 15 min. We determined protein concentrations by BCA (bicinchoninic acid) Protein Assay (Pierce, Rockford, IL). We ran samples (20 or 50 μg) on 10% Tris-HCl sodium dodecyl sulfate polyacrylamide gels (Bio-Rad, Hercules, CA) and transferred them to poly-vinyl difluoride membranes (Immobilon, Millipore, Milford, MA). We stained transferred membranes with Ponceau (Sigma) and scanned them with Canoscan LiDE70 (Canon, Lake Success, NY). Membranes were blocked in 5% bovine serum albumin with shaking for 1 hr at room temperature and incubated overnight at 4°C with IL1b rabbit polyclonal antibody (Abcam, Cambridge, MA), tumor necrosis factor-α (TNF-α) rabbit polyclonal antibody (Peprotech, Rocky Hill, NJ), inhibitor of nuclear factor κBα (IκBα) rabbit polyclonal antibody (Cell Signaling, Beverly, MA), Traf6 rabbit monoclonal antibody (Abcam), and Myd88 rabbit polyclonal antibody (Abcam), each followed by goat anti-rabbit IgG conjugated to horseradish peroxidase (Santa Cruz Biotechnology, Inc., Santa Cruz, CA) for 1 hr at room temperature. Blots were developed using Super Signal Pico Substrate (Pierce) and imaged on X-Omat Blue XB-1 Scientific Imaging Film (Kodak, Rochester, NY). We quantified and averaged two representative Ponceau bands (of highly expressed, As-unaffected proteins with molecular weights distinct from any of the As-affected proteins of interest) to serve as loading control. We performed densitometry using Scion Imaging Software (Scion Corp., Frederick, MD).

### Statistical analysis

We performed statistical analysis with GraphPad Prism (version 5.0a for Macintosh; GraphPad Software Inc., La Jolla, CA) using an unpaired *t*-test with 95% confidence interval. We used one-tailed *t*-tests for quantitative real-time PCR confirmation of microarray results, and two-tailed *t*-tests for immunoblot densitometry analysis.

## Results

**Figure 1:**
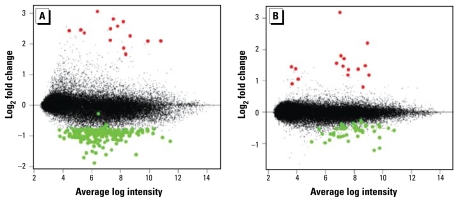
Chronic As exposure at the current drinking water standard alters gene expression in mouse lung: intensity-dependent data from the microarray analysis of mice exposed to 10 ppb As (*A*) or 100 ppb As (*B*) in drinking water compared with control. Genes identified by rank-product analysis to be significantly up-regulated by As exposure are highlighted in red, and genes identified to be significantly down-regulated are highlighted in green.

Animals were exposed to As in food or drinking water with a background of AIN-76A semipurified, defined diet, as described in “Materials and Methods.” Analysis of total As levels in the tails of mice receiving 0, 10, or 100 ppb As in drinking water revealed a proportional, dose-dependent increase in As body burden with these treatments (data not shown). To identify differentially expressed genes between the control and As-exposed groups, we used rank-product analysis (Breitling et al. 2004) with a *p*-value of 0.05. Figure 1 represents an “MA plot,” which shows the intensity-dependent fold-change ratios (log_2_) of each probe set (each represented by a black dot) from the microarray data for the animals exposed to 10 ppb As (Figure 1A) or 100 ppb As (Figure 1B) in the drinking water, compared with animals receiving no As in their drinking water. One sample in the 100-ppb As exposure group showed far more RNA degradation according to the AffyRNADeg program (a function available from BioConductor as part of the Affymetrix library; http://www.affymetrix.com). Including this sample would have caused an intensity-dependent systematic error consistent with degradation, so it was excluded from further analysis. Genes identified by rank-product analysis to be significantly up- or down-regulated compared with control are highlighted in red or green, respectively. We observed obvious differences in expression patterns for both levels of exposure, but there was an evident dominance of down-regulation of many transcripts with the 10-ppb As exposure group, as identified by rank-product analysis. Several of the probes appeared to be increased on the MA plot, but rank-product analysis did not identify these as significant because the mean results were being influenced by one animal in the group. In subsequent analyses, we focused principally on the changes observed in the drinking-water groups, although the group of animals exposed to 10 ppb As added to the food had mRNA alterations similar to those of the animals that received 10 ppb As in drinking water. Analysis of the rank-product lists revealed changes in the expression of many groups of genes with functions in cell adhesion and migration, membrane channels, various receptors, differentiation and proliferation markers, and, most strikingly, the immune response [Supplemental Material, Table 1 (http://www.ehponline.org/members/2009/0800199/suppl.pdf)].

**Figure 2:**
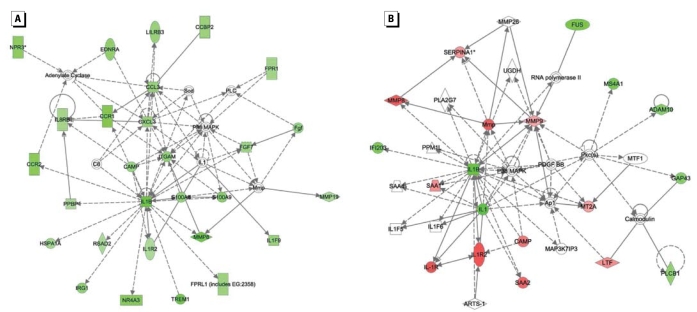
IPA of gene expression changes induced by As exposure indicates similar networks for both the 10-ppb exposure (*A*) and 100-ppb exposure (*B*). The top functions of these networks are cellular movement, hematologic system development and function, and immune response. Gene symbols are located in the center of each box; green shapes indicate down-regulated genes, and red shapes indicate up-regulated genes, compared with control. Nonshaded shapes indicate genes that IPA inserted to obtain the most connections within the network, but these genes were not regulated by the experimental treatment. See Supplemental Material, Table 4 (http://www.ehponline.org/members/2009/0800199/suppl.pdf) for legend to symbols.

We then used the lists generated by rank-product analysis [Figure 1; Supplemental Material, Table 1 (http://www.ehponline.org/members/2009/0800199/suppl.pdf)] to assess network changes with IPA. A similar IPA network of genes was regulated in both the 10-ppb and 100-ppb drinking-water As exposures, with the top associated biological functions being cellular movement, hematologic system development and function, and immune response. At the 10-ppb dose (Figure 2A), the transcripts were largely decreased (green shading) compared with control, whereas at the 100-ppb dose (Figure 2B) many transcripts were increased (red shading) compared with control. Both of these networks centered around the inflammatory cytokine *Il1b*, which was down-regulated in both As exposures.

**Figure 3:**
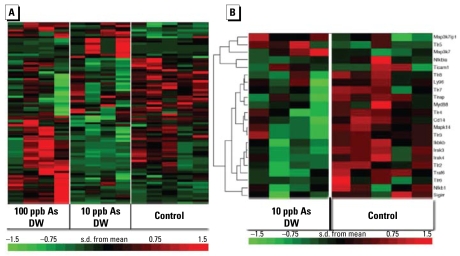
Genomewide (*A*) and TLR/IL-1 pathway (*B*) transcriptome microarray analysis of immune response genes. DW, drinking water. Each column represents a separate animal, grouped manually by treatment; genes are clustered by relational analysis using standard Pearson correlation (see “Materials and Methods”). Individual Affymetrix probes that were differentially expressed are represented across each row. Green boxes indicate genes that were significantly down-regulated, and red boxes indicate genes that were up-regulated, relative to control. (*A*) Heat map of clustered, differentially regulated probes. The ANOVA function of R was used to generate a list of the differentially regulated genes. A comprehensive list of genes known to have a function in the immune response was intersected with a list of the top 5% of genes identified by ANOVA. (*B*) Heat map of raw RMA normalized probe data based in literature references used to create a list of genes known to play a role in the TLR/IL1R signaling pathway.

Based on these alterations in immune-related gene expression, we investigated the changes in this subset of genes within the microarray data in more detail, first using other statistical methods such as analysis of variance (ANOVA), to aid in the detection of more detailed expression patterns. We searched Affymetrix NETAFFX for probe descriptions that included “immune response,” generating a comprehensive list of 973 probe identifiers. We then intersected this list of immune probes (both innate and adaptive responses) with a list of the top 5% altered genes as identified by ANOVA. The heat map in Figure 3A represents the results and largely shows the same trend, particularly a down-regulation in response to 10 ppb As, previously identified with the rank-product lists in IPA.

Careful analysis of the differentially regulated immune genes revealed that several of these genes are involved in the innate immune response, particularly IL-1 and TLR signaling. *Il1r1* shares a homology domain with the TLR family, and the activation of both these receptors induces a complex signaling pathway ultimately resulting in the induction of genes involved in the immune response, such as *Il1b*. We constructed a list of key molecules in the IL-1/TLR signaling pathway from literature references; Figure 3B shows the raw RMA (robust multiarray) normalized microarray data in the heat map for animals exposed to 10 ppb As in drinking water for 5 weeks (Figure 3B). We observed a clear down-regulation in this specific pathway for the As-exposed animals. Supplemental Material, Table 2 (http://www.ehponline.org/members/2009/0800199/suppl.pdf) gives details for the list of genes represented in Figure 3A.

**Figure 4:**
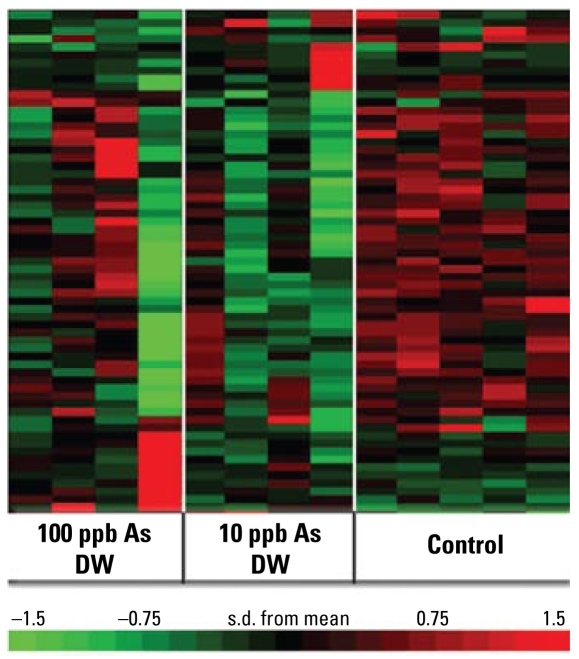
Genomewide transcriptome microarray analysis of cellular migration genes: heat map of clustered, differentially regulated probes. DW, drinking water. Genomewide transcriptome microarrays were run and treated as described in Figure 3. The ANOVA function of R was used to create a list of the differentially regulated genes. A comprehensive list of genes known to have a function in the cellular migration was created using Affymetrix NETAFFX. This list was intersected with a list of the top 20% of genes identified by ANOVA.

Numerous molecules involved in cellular adhesion and migration were present on the rank-product gene list, and the cellular movement function was identified by IPA to be affected by As exposure. We also investigated the changes within this pathway using other statistical methods. We searched Affymetrix NETAFFX for “GO [gene ontology] biological functions” that included “cellular migration.” This generated a list of 359 probe identifiers, which we intersected with a list of the top 20% of altered genes as identified by ANOVA. The heat map representing these results (Figure 4) clearly demonstrates a difference in the gene expression pattern between the control and As-exposed animals. Many of these genes play integral roles in migration/adhesion events and/or cellular structural integrity, such as vinculin, laminin, platelet/endothelial cell adhesion molecule 1 (*PECAM1*), *Rac1*, and a number of integrins. Supplemental Material, Table 3 (http://www.ehponline.org/members/2009/0800199/suppl.pdf) gives details for the list of genes represented in Figure 4.

**Figure 5:**
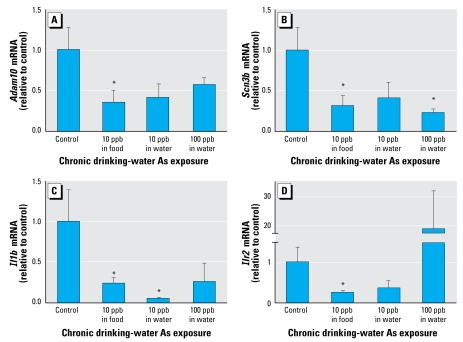
PCR confirmation of genes identified by microarray analysis to be altered by As exposure: transcript levels for *Adam10* (*A*), *Scn3b* (*B*), *Il1b* (*C*), and *Il1r2* (*D*) determined by quantitative real-time RT-PCR. Relative mRNA levels are expressed as arbitrary units, normalized to the data from control animals. Each bar represents mean + SEM of relative values from 4–6 animals per treatment. *p < 0.05 versus control (one-tailed Student’s *t*-test).

We performed confirmatory quantitative RT-PCR on a select number of genes to confirm the changes observed by the microarray (Figure 5). Because we observed changes in adhesion and migration pathways and a number of intracellular channels, we confirmed the transcript levels for *Adam10* (a disintegrin and metalloprotease domain 10; Figure 5A) and *Scn3b* (voltage-gated sodium channel subunit 3 beta; Figure 5B). We also confirmed the mRNA levels for *Il1b* (Figure 5C) and the IL-1 decoy receptor *Il1r2* (Figure 5D) by RT-PCR. Not all changes were statistically significant because of variability in the absolute expression among the animals of the groups. However, several were significant (*p* < 0.05), and the remainder showed strong trends in the directions expected from the statistically significant microarray data.

**Figure 6:**
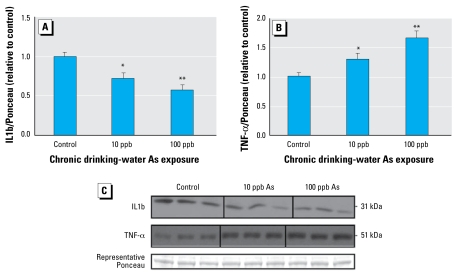
Low-dose As alters protein levels of IL1b and TNF-α in whole lung homogenate: densitometric quantification from all animals from two separate experimental repeats (*n* = 6 per experimental repeat; *n* = 12 total) in each group for levels of IL1b (*A*) and TNF-α (*B*) protein (representative Western blot results for three mice from each treatment group). (*C*) Data from two representative Ponceau bands averaged and used for normalization. Error bars represent mean + SEM of values for 12 mice per treatment. *p < 0.05, **p < 0.01, versus control, two-tailed Student’s *t*-test.

We also investigated alterations at the protein level for inflammatory cytokines and signaling molecules. Because of the limited quantity of tissue remaining from the microarray studies, we repeated the chronic As exposures, optimizing for protein collection, in two separate studies. We used immunoblot to detect levels of pro-IL1b in whole lung homogenate. IL1b propeptide was significantly decreased at both levels of drinking-water As exposure (Figure 6A,C), coincident with the mRNA levels (Figure 5C). We also investigated the levels of TNF-α, another important inflammatory cytokine. Although we did not observe any alterations in TNF-α mRNA levels in the microarray data, we found a significant increase in homotrimeric 51-kDa TNF-α protein levels at both the 10- and 100-ppb drinking-water As exposures (Figure 6B,C).

**Figure 7:**
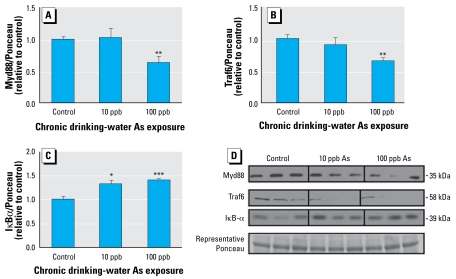
Low-dose As alters the protein levels of TLR and IL1R signaling pathway mediators: densitometric quantification from all animals from two separate experimental repeats (*n* = 6 per experimental repeat; *n* = 12 total) in each group for Myd88 (*A*), Traf6 (*B*), and IκBα (*C*) protein levels (representative Western blot results for three mice from each treatment group). (*D*) Data from two representative Ponceau bands were averaged and used for normalization. Error bars represent mean + SEM of values for 12 mice per treatment. *p < 0.05, **p < 0.01, *** *p* < 0.001, versus control, two-tailed Student’s *t*-test.

Based on the observations in the microarray data, we also investigated the protein levels of key components of the TLR/IL1R signaling pathways, which includes Myd88, Traf6, and IκBα. The array results indicated that Myd88 (a TLR/IL1R adaptor molecule) and Traf6 (TNF receptor superfamily and TLR/IL1R family signaling mediator) were decreased by As exposure as low as 10 ppb, but the microarray studies did not indicate a change in IκBα. Myd88 and Traf6 protein levels were significantly decreased at the 100-ppb drinking-water As exposure (Figure 7A,B,D). Although a number of the animals exposed to 10 ppb As had changes similar to those with 100-ppb exposures, the overall effect was not significant. Ultimately, induction of many inflammatory genes takes place in a nuclear factor κB (NFκB)–dependent manner. Because As has been shown to affect NFκB signaling, we investigated the protein levels of IκBα, the NFκB inhibitory protein. We found IκBα protein levels to be significantly increased at both doses of As exposure (Figure 7C,D).

**Figure 8:**
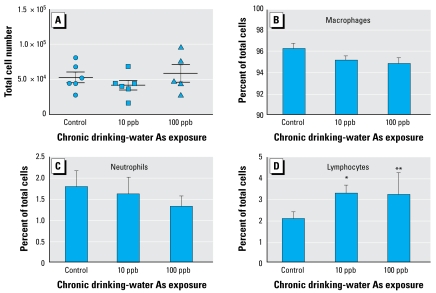
Low-dose As exposure increases lymphocytes in BALF. (*A*) Total cells recovered from the lungs of control and As-exposed animals by bronchoalveolar lavage. Cell differentials were determined by cellular morphology from 10 random fields after staining of cytospin slides. Macrophages (*B*), neutrophils (*C*), and lymphocytes (*D*) were identified (represented as a percentage of total cells). Error bars represent mean + SEM of values for five to six mice per treatment. *p < 0.05, **p < 0.01, versus control, two-tailed Student’s *t*-test.

Because we obtained these results from whole lung homogenate, one explanation could be an alteration in the number and percentage of immune cell populations in the lung. We have previously reported that chronic As exposure at similar doses did not induce any histologic changes or overt cell population changes in mouse lung tissue sections (Andrew et al. 2007). To confirm these results, we investigated total and differential cell counts into the BALF of the As-exposed animals. We found that As exposure did not significantly change the total cell counts in BALF (Figure 8A) or the percentages of macrophages (Figure 8B) or neutrophils (Figure 8C). We observed a trend toward decreased macrophage and neutrophil percentages in the As-exposed animals, but the difference was not significant. However, we did observe small but significant increases in the percentage of lymphocytes in the lavage fluid of the animals exposed to 10 and 100 ppb As (Figure 8D).

## Discussion

In this study, we confirmed several significant alterations in gene expression by transcriptome profiling in the mouse lung after chronic low-dose (10 and 100 ppb) As exposure. In addition, the expression profiles of the animals revealed numerous changes in the mRNA levels of genes involved with cell adhesion and migration, various cellular channels and receptors, differentiation and proliferation, and, most remarkably, the immune response, which was the dominant group of affected genes in this analysis. Interestingly, gene expression changes for a similar list of inflammatory mediators were recently identified by microarray analysis of newborn cord blood in a population of As-exposed mothers (Fry et al. 2007).

Chronic exposure to As in the drinking water has been associated with an increased risk of lung disease in exposed human populations (Ghosh et al. 2007; Marshall et al. 2007; Zaldivar and Ghai 1980). The lung appears to be uniquely sensitive to As, in that chronic ingestion of relatively low, nonovertly toxic doses can elicit increased disease risk, whereas other lung toxicants require inhalation to elicit similar disease risks. Nonmalignant lung disease risk due to As exposure includes bronchiectasis and decreased pulmonary function (Mazumder 2007; Smith et al. 2006), and evidence suggests that the mechanism behind As-related lung disease may be altered inflammatory responses as opposed to direct toxicity (De et al. 2004). Recent laboratory studies in mice have indicated that environmentally relevant levels of As in drinking water can induce alterations in lung gene and protein expression (Andrew et al. 2007; Lantz et al. 2007). In a separate study, we previously reported that some of these gene alterations included genes associated with immune function (Andrew et al. 2007).

Interestingly, chronic ingestion of 10 ppb As in either food or water produced similar patterns and magnitudes of gene expression change, suggesting that As contamination of food as well as water may pose lung disease risks. At the 10-ppb drinking-water dose, many of the changes observed in the microarray, particularly with respect to the immune response, were decreases in transcript levels compared with control. In particular, genes involved in cell migration, hematologic system development and function, and immune response were significantly altered, as identified by rank-product and IPA analyses. IPA-reported connections among genes are not necessarily direct protein–protein interactions but rather represent a variety of reported connections based on published scientific literature. However, such networks can reveal interesting patterns that do not represent canonical pathways per se.

As we and others have observed in previous studies, in this analysis we observed that As elicits noncanonical dose responses at the gene expression level, where some genes were commonly affected at different doses but in opposite directions, whereas other genes were part of dose-specific sets that were nonover-lapping at different doses (Andrew et al. 2003, 2007; Bodwell et al. 2004, 2006; Davey et al. 2007, 2008; Kamat et al. 2005)—something we have referred to as phenotypic switching. This switching can occur at two doses that differ by as little as 5- to 10-fold, and in this study we observed this in comparing gene expression changes at 10 and 100 ppb As in drinking water.

The patterns of gene expression changes we observed led us to confirm a number of genes by quantitative PCR, which demonstrated that many of these individual mRNA changes were even greater than the microarray indicated, something that commonly has been observed in such studies, which suggests that the quantitative range of microarrays for individual genes is much narrower than the highly quantitative and gene-specific quantitative RT-PCR method.

The role for Adam10 in As toxicity is interesting because ADAM family members are proteolytic enzymes, functioning in “protein ectodomain shedding” and play crucial roles in many signaling events (Blobel 2005). It has been recently reported that As exposure at a similar dose can increase MMP-9 production and decrease wound healing/cellular migration in human airway epithelial cells (Olsen et al. 2008). Adam10 has been shown to be a critical component of epithelial cell–cell adhesion and migration (Maretzky et al. 2005). The changes we observed in cellular migration and adhesion genes, including Adam10, concur with recent reports that As can alter focal adhesions and decrease cellular migration (Yancy et al. 2005) and wound healing *in vitro* (Olsen et al. 2008).

Changes in mRNA expression are a powerful tool for investigating effects of toxicants and can reveal detailed pathway information using whole genome transcriptome profiling. However, biological effects of most genes are elicited by their protein products, and changes in mRNA steady-state expression sometimes correlate poorly with changes in protein expression or function. Thus, we were interested in whether there were measurable changes in expression of key immune response genes at the protein expression level in these animals. Quantitative Western blotting revealed significant, dose-dependent changes in the levels of a number of important innate immune proteins.

The alterations in the regulation of many immune genes, especially *Il1b* and *TNF*-α, were particularly striking in the As-exposed animals and indicated a potentially important mechanism of As-related disease in the lung. IL1b is an inflammatory cytokine that can exert effects on nearly every cell type, with numerous functions, including increasing the expression of cyclooxygenase and inducible nitric oxide synthase, other cytokines, chemokines, adhesion molecules, tissue proteases, and matrix metalloproteases (Dinarello 2005). We found many genes in the aforementioned category that were altered by As exposure [Supplemental Material, Table 1(http://www.ehponline.org/members/2009/0800199/suppl.pdf)]. TNF-α is another inflammatory cytokine that plays a key role in regulating the innate immune response. TNF-α production, like IL1b, is tightly regulated by mRNA stability and translation silencing. The intricate feedback loops that regulate TNF expression help to control the period and degree of TNF-α production after the initial induction (Schottelius et al. 2004). It is unclear why TNF-α and IL1b are affected in opposite directions; however, it is well recognized that although TNFα and IL1b are both inflammatory cytokines, they are multifunctional and signal through different pathways and receptors (TNF receptor 1 and 2 vs. IL1R). One explanation is that the increases in TNF-α result from the increased number of lymphocytes within the lung. An alternative is that the increases in TNF-α result from the role of TNF-α in another pathway, such as mitogen-activated protein kinase (MAPK) or death signaling pathways, as opposed to NFκB inflammatory cytokine signaling.

The observed alterations in key components of IL-1/TLR signaling pathways suggest that this signaling pathway is an important target of chronic As exposure in the lung. IκBα was increased at both levels of exposure. Myd88 and Traf6 were significantly decreased at the 100-ppb dose. TLRs recruit a series of adaptor molecules, ultimately leading to a signaling cascade that activates the transcription factor NFκB. Myd88 is an adaptor protein in this process (Akira and Takeda 2004). Traf6 is unique adaptor in its ability to transduce signals from both TNF receptor and IL1R/TLR pathways (Wu and Arron 2003). As exposure (in the form of As trioxide) has been previously reported to increase IκBα levels in the whole lung homogenate in a murine model of asthma (Zhou et al. 2006). Several groups have reported alterations in IκBα levels as a result of As exposure, but the direction and specifics of the response appear to depend on the dose, time, and cell type. Our results show that chronic As exposure affects the gene and protein regulation of this important signaling pathway *in vivo*, primarily in a suppressive manner.

Finally, given that macrophage and neutrophil populations were unchanged in the BALF of the As-exposed animals, the observations in the study indicate that the changes in gene and protein expression are attributable to an effect of As on innate immune signaling pathways, as opposed to a change in innate immune cell populations within the lung. The observed increase of lymphocytes was unexpected and warrants future investigation but may ultimately explain the increased levels of TNF-α. Under inflammatory conditions, macrophages are the primary producers of TNF-α, but lymphocytes can also be potent producers of TNF-α. Because very few lymphocytes can be isolated from the lungs of mice maintained in a sanitary animal facility without introducing an intentional infection, the observed increase in lymphocyte populations within the lung will be investigated in a different model system.

Overall, this study indicates that chronic low-dose As exposure can have significant effects on expression profiles in mouse lung and, more important, on the protein levels of many important immune mediators at doses as low as the current U.S. EPA drinking-water standard. The effect of chronic low-dose As on the regulation of inflammatory pathways indicates a potential mechanism of As-related disease risk in the lung. After 5 weeks of low-dose exposure, the animals displayed no signs of overt toxicity or a physiologic phenotype, as might be expected at such a low dose for a relatively short period of time. However, the observed down-regulation of key innate immune regulators indicates that As-exposed animals may be at an increased risk of contracting respiratory infection. This may be a potential mechanism behind the increased risk of lung disease in As-exposed populations, such as bronchiectasis, which is known to be related to an increased incidence of respiratory infection. Because the TLR/IL-1 pathway plays such a critical function in the initial response to pathogens, we predict that there will be a functional consequence as a result of the changes we observed. A recent study has indicated that in response to an ovalbumin challenge (a well-accepted murine model of allergic asthma), mice treated with arsenic trioxide demonstrated significant immune suppression indicated by decreases in cytokine production, reduced eosinophil recruitment/migration to the lung, and diminished NFκB activation (Zhou et al. 2006). It has also recently been shown that *in utero* exposure to As in human populations is positively correlated with acute respiratory infections (Raqib et al. 2009). Future studies will aim to address whether the observed dysregulation by chronic low-dose As exposure can lead to the development of a physiologic phenotype or altered disease risk in response to respiratory viral infection.

## References

[b1-ehp-117-1108] Abernathy CO, Liu YP, Longfellow D, Aposhian HV, Beck B, Fowler B (1999). Arsenic: health effects, mechanisms of actions, and research issues. Environ Health Perspect.

[b2-ehp-117-1108] Agency for Toxic Substances and Disease Registry (1999). Toxicological Profile for Arsenic (Update).

[b3-ehp-117-1108] Akira S, Takeda K (2004). Toll-like receptor signalling. Nat Rev Immunol.

[b4-ehp-117-1108] Andrew AS, Bernardo V, Warnke LA, Davey JC, Hampton T, Mason RA (2007). Exposure to arsenic at levels found in U.S. drinking water modifies expression in the mouse lung. Toxicol Sci.

[b5-ehp-117-1108] Andrew AS, Jewell DA, Mason RA, Whitfield ML, Moore JH, Karagas MR (2008). Drinking-water arsenic exposure modulates gene expression in human lymphocytes from a U.S. population. Environ Health Perspect.

[b6-ehp-117-1108] Andrew AS, Warren AJ, Barchowsky A, Temple KA, Klei L, Soucy NV (2003). Genomic and proteomic profiling of responses to toxic metals in human lung cells. Environ Health Perspect.

[b7-ehp-117-1108] Blobel CP (2005). ADAMs: key components in EGFR signalling and development. Nat Rev.

[b8-ehp-117-1108] Bodwell JE, Gosse JA, Nomikos AP, Hamilton JW (2006). Arsenic disruption of steroid receptor gene activation: complex dose-response effects are shared by several steroid receptors. Chem Res Toxicol.

[b9-ehp-117-1108] Bodwell JE, Kingsley LA, Hamilton JW (2004). Arsenic at very low concentrations alters glucocorticoid receptor (GR)-mediated gene activation but not GR-mediated gene repression: complex dose-response effects are closely correlated with levels of activated GR and require a functional GR DNA binding domain. Chem Res Toxicol.

[b10-ehp-117-1108] Breitling R, Armengaud P, Amtmann A, Herzyk P (2004). Rank products: a simple, yet powerful, new method to detect differentially regulated genes in replicated microarray experiments. FEBS Lett.

[b11-ehp-117-1108] Breitling R, Herzyk P (2005). Rank-based methods as a non-parametric alternative of the T-statistic for the analysis of biological microarray data. J Bioinform Comput Biol.

[b12-ehp-117-1108] Corsini E, Asti L, Viviani B, Marinovich M, Galli CL (1999). Sodium arsenate induces overproduction of interleukin-1alpha in murine keratinocytes: role of mitochondria. J Invest Dermatol.

[b13-ehp-117-1108] Davey JC, Bodwell JE, Gosse JA, Hamilton JW (2007). Arsenic as an endocrine disruptor: effects of arsenic on estrogen receptor-mediated gene expression *in vivo* and in cell culture. Toxicol Sci.

[b14-ehp-117-1108] Davey JC, Nomikos AP, Wungjiranirun M, Sherman JR, Ingram L, Batki C (2008). Arsenic as an endocrine disruptor: arsenic disrupts retinoic acid receptor- and thyroid hormone receptor-mediated gene regulation and thyroid hormone-mediated amphibian tail metamorphosis. Environ Health Perspect.

[b15-ehp-117-1108] De BK, Majumdar D, Sen S, Guru S, Kundu S (2004). Pulmonary involvement in chronic arsenic poisoning from drinking contaminated ground-water. J Assoc Phys India.

[b16-ehp-117-1108] Dinarello CA (2005). Interleukin-1beta. Crit Care Med.

[b17-ehp-117-1108] Fry RC, Navasumrit P, Valiathan C, Svensson JP, Hogan BJ, Luo M (2007). Activation of inflammation/NF-kappaB signaling in infants born to arsenic-exposed mothers. PLoS Genet.

[b18-ehp-117-1108] Ghosh P, Banerjee M, De Chaudhuri S, Chowdhury R, Das JK, Mukherjee A (2007). Comparison of health effects between individuals with and without skin lesions in the population exposed to arsenic through drinking water in West Bengal, India. J Expo Sci Environ Epidemiol.

[b19-ehp-117-1108] Gosse J, Hampton T, Davey J, Hamilton J, Sahu S (2008). A new approach to analysis and interpretation of toxicogenomic gene expression data and its importance in examining biological responses to low, environmentally relevant doses of toxicants. Toxicogenomics: A Powerful Tool for Toxicity Assessment.

[b20-ehp-117-1108] Kamat CD, Green DE, Curilla S, Warnke L, Hamilton JW, Sturup S (2005). Role of HIF signaling on tumorigenesis in response to chronic low-dose arsenic administration. Toxicol Sci.

[b21-ehp-117-1108] Karagas MR, Stukel TA, Tosteson TD (2002). Assessment of cancer risk and environmental levels of arsenic in New Hampshire. Int J Hyg Environ Health.

[b22-ehp-117-1108] Kozul CD, Nomikos AP, Hampton TH, Warnke LA, Gosse JA, Davey JC (2008). Laboratory diet profoundly alters gene expression and confounds genomic analysis in mouse liver and lung. Chem-Biol Interact.

[b23-ehp-117-1108] Lage CR, Nayak A, Kim CH (2006). Arsenic ecotoxicology and innate immunity. Integr Comp Biol.

[b24-ehp-117-1108] Lantz RC, Lynch BJ, Boitano S, Poplin GS, Littau S, Tsaprailis G (2007). Pulmonary biomarkers based on alterations in protein expression after exposure to arsenic. Environ Health Perspect.

[b25-ehp-117-1108] Lemarie A, Morzadec C, Bourdonnay E, Fardel O, Vernhet L (2006). Human macrophages constitute targets for immunotoxic inorganic arsenic. J Immunol.

[b26-ehp-117-1108] Maretzky T, Reiss K, Ludwig A, Buchholz J, Scholz F, Proksch E (2005). ADAM10 mediates E-cadherin shedding and regulates epithelial cell-cell adhesion, migration, and beta-catenin translocation. Proc Natl Acad Sci USA.

[b27-ehp-117-1108] Marshall G, Ferreccio C, Yuan Y, Bates MN, Steinmaus C, Selvin S (2007). Fifty-year study of lung and bladder cancer mortality in Chile related to arsenic in drinking water. J Natl Cancer Inst.

[b28-ehp-117-1108] Mazumder DN (2007). Arsenic and non-malignant lung disease. J Environ Sci Health.

[b29-ehp-117-1108] National Research Council (1999). Arsenic in Drinking Water.

[b30-ehp-117-1108] Nayak AS, Lage CR, Kim CH (2007). Effects of low concentrations of arsenic on the innate immune system of the zebrafish (*Danio rerio*). Toxicol Sci.

[b31-ehp-117-1108] Olsen CE, Liguori AE, Zong Y, Lantz RC, Burgess JL, Boitano S (2008). Arsenic upregulates MMP-9 and inhibits wound repair in human airway epithelial cells. Am J Physiol.

[b32-ehp-117-1108] Patterson R, Vega L, Trouba K, Bortner C, Germolec D (2004). Arsenic-induced alterations in the contact hypersensitivity response in Balb/c mice. Toxicol Appl Pharmacol.

[b33-ehp-117-1108] Raqib R, Ahmed S, Sultana R, Wagatsuma Y, Mondal D, Hoque AM (2009). Effects of *in utero* arsenic exposure on child immunity and morbidity in rural Bangladesh. Toxicol Lett.

[b34-ehp-117-1108] Schottelius AJ, Moldawer LL, Dinarello CA, Asadullah K, Sterry W, Edwards CK (2004). Biology of tumor necrosis factor-alpha- implications for psoriasis. Exp Dermatol.

[b35-ehp-117-1108] Selgrade MK (2007). Immunotoxicity: the risk is real. Toxicol Sci.

[b36-ehp-117-1108] Smith AH, Hopenhayn-Rich C, Bates MN, Goeden HM, Hertz-Picciotto I, Duggan HM (1992). Cancer risks from arsenic in drinking water. Environ Health Perspect.

[b37-ehp-117-1108] Smith AH, Marshall G, Yuan Y, Ferreccio C, Liaw J, von Ehrenstein O (2006). Increased mortality from lung cancer and bronchiectasis in young adults after exposure to arsenic *in utero* and in early childhood. Environ Health Perspect.

[b38-ehp-117-1108] Soto-Pena GA, Luna AL, Acosta-Saavedra L, Conde P, Lopez-Carrillo L, Cebrian ME (2006). Assessment of lymphocyte subpopulations and cytokine secretion in children exposed to arsenic. FASEB J.

[b39-ehp-117-1108] Tapio S, Grosche B (2006). Arsenic in the aetiology of cancer. Mutat Res.

[b40-ehp-117-1108] U.S. EPA (2001). National primary drinking water regulations; arsenic and clarifications to compliance and new source contaminants monitoring. Fed Reg.

[b41-ehp-117-1108] Vega L, Styblo M, Patterson R, Cullen W, Wang C, Germolec D (2001). Differential effects of trivalent and pentavalent arsenicals on cell proliferation and cytokine secretion in normal human epidermal keratinocytes. Toxicol Appl Pharmacol.

[b42-ehp-117-1108] Watanabe C, Inaoka T, Matsui T, Ishigaki K, Murayama N, Ohtsuka R (2003). Effects of arsenic on younger generations. J Environ Sci Health.

[b43-ehp-117-1108] Wu H, Arron JR (2003). TRAF6, a molecular bridge spanning adaptive immunity, innate immunity and osteoimmunology. Bioessays.

[b44-ehp-117-1108] Wu MM, Chiou HY, Ho IC, Chen CJ, Lee TC (2003). Gene expression of inflammatory molecules in circulating lymphocytes from arsenic-exposed human subjects. Environ Health Perspect.

[b45-ehp-117-1108] Yancy SL, Shelden EA, Gilmont RR, Welsh MJ (2005). Sodium arsenite exposure alters cell migration, focal adhesion localization and decreases tyrosine phosphorylation of focal adhesion kinase in H9C2 myoblasts. Toxicol Sci.

[b46-ehp-117-1108] Yu HS, Chang KL, Yu CL, Wu CS, Chen GS, Ho JC (1998). Defective IL-2 receptor expression in lymphocytes of patients with arsenic-induced Bowen’s disease. Arch Dermatol Res.

[b47-ehp-117-1108] Zaldivar R, Ghai GL (1980). Clinical epidemiological studies on endemic chronic arsenic poisoning in children and adults, including observations on children with high- and low-intake of dietary arsenic. Zentralbl Bakteriol.

[b48-ehp-117-1108] Zhou LF, Zhu Y, Cui XF, Xie WP, Hu AH, Yin KS (2006). Arsenic trioxide, a potent inhibitor of NF-kappaB, abrogates allergen-induced airway hyperresponsiveness and inflammation. Respir Res.

